# Impact of regulatory capital on bank interest margins: Moderating role of default risk

**DOI:** 10.1016/j.heliyon.2024.e30554

**Published:** 2024-05-01

**Authors:** Munni Begum, Mohammed Mizanur Rahman, Mohammad Omar Faruq

**Affiliations:** a Department of Accounting and Finance, North South University, Dhaka, Bangladesh; b Department of AIS, Comilla University, Cumilla, 3506, Bangladesh

**Keywords:** Bank interest margin, GMM estimation, Regulatory capital, Default risk, financial crisis

## Abstract

The recent pandemic and aftermath debate regarding bank interest margins deserve special attention and have become policy dialogue in emerging economies. However, the previous literature's findings were largely inconclusive and ignored influential variables such as the impact of default risk on bank interest margins. Using a two-step system GMM estimation considering 32 Bangladeshi commercial banks from 2000 to 2022, we produce robust evidence that higher regulatory capital restrictions reduce the bank interest margin, while increased default risk induces the bank interest margin. The impact intensity during the COVID pandemic is higher than in the pre-COVID period. Moreover, we find the synergy effect of regulatory capital and default risk assists in reducing the bank interest margin. Bank margin persistently fell during the capital market crash period, whereas it rose in the financial crisis period. We cast several robustness tests to confirm our main findings. These findings could generate important implications for bank stakeholders and policymakers.

## 1 Introduction

With the tragic fall of the world economy in 2007–2008 and after that, the banking sector stability is an important issue. The crisis startled the world economy at large and was the most significant one over the past few decades [1].

This historical adverse shock raises the question about the effectiveness of risk-based capital regulation. Notably, the importance of bank interest margins, risk-taking, and capital regulation research has grown in recent years in reply to the global financial crisis, which started from the infamous Lehman Brothers in the USA and spread worldwide [2]. This global financial crisis helps us focus on the consequences of the failure of the banking system on an economy [3]. The major learning from this recent financial tsunami was that the present regulatory frameworks were insufficient to protect banks from excessive risk-taking [4]. Moreover, the recent banking crisis also reflects the volatility of the banking nature & the tendency of risk-seeking [5]. Further, the current credit crunch helps to understand the factors affecting bank risk-taking in the lower bank capital environment [6].

The second shock through the recent pandemic (COVID-19) stacks the world economy at a point [7].

The Basel Accord is a series of structures that have been taken to maintain the banking system's stability by reducing systematic risk [8]. The Basel Committee on Banking Supervision (BCBS) formulated the first Basel Accord guideline in 1988 [9]. Basel I mainly focus on minimum capital requirements and bank credit risk, asserting mechanisms to control assets portfolio risk. By following this, Bangladesh Bank (BB)^1^ adopted the first Basel Accord guidelines in 1996 in the Bangladesh banking sector. Due to Basel I's shortfalls, BCBS introduced Basel II in 2004 to make risk-based capital regulation more efficient [9].

Up to the fourth survey of the World Bank (WB),^2^ 97 countries in the world implemented full phase Basel II in their banking sector [10], whereas other countries are planning to adopt it after the recent global financial crisis (GFC).

As before, the regulatory authority of Bangladesh was unable to adopt Basel II in time; consequently, it was adopted in 2007. The regulatory authority follows observation first and applies later in the financial sector [11]. Moreover, the absence of related experts and timeliness are the important backdrops for the delay in the adaption of Basel II. Thus, the application analysis of Basel guidelines in Bangladeshi banking becomes urgent once again.

Bangladesh's banking is divided into two major categories: Islamic and conventional banking [12].

In terms of Basel Accord capital requirements, Islamic banks are more responsive than conventional [13]. Islamic banks face a different risk than conventional banking: ' fiduciary risk', which arises from the client's trust and beliefs [14]. This study considers six Islamic commercial banks out of 32 sample banks. According to the Basel III implementation status report by Bangladesh Securities and Exchange Commission (BSEC),^3^ Islamic banks hold higher tier-I capital than conventional banking in Bangladesh.

**Fig. 1:**
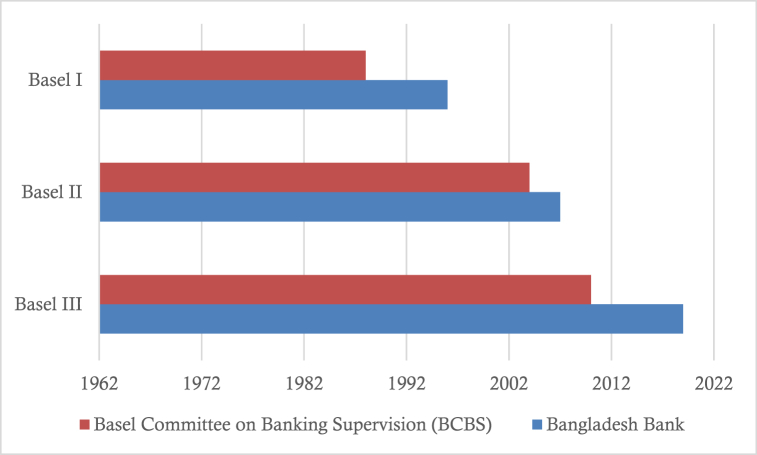
Implementation status of Basel Accords.

Basel III was issued by BCBS in November 2010, which had to apply in banking in 2015 [15], whereas in Bangladeshi banking, it applied in 2019 due to the late adaptation of Basel II. An overview of the implementation status can be found in Fig. 1. However, the updated Basel III capital requirement is not out of dispute. Later, on March 27, 2020, the Basel Committee on Banking Supervision (BCBS) announced its deferral of implementing the final Basel III package (Basel IV) by one year to 2023 in response to COVID-19 [16].

Amid the COVID-19 pandemic, the central banks in developed economies showcased swift and forceful reactions using the full deployment of available crisis management tools [17].

Though the regulatory capital restrictions are likely to defend the future banking industry failure caused by the financial crisis [18,19], however, the regulatory restrictions are not out of criticism. In specific, the influence of regulatory capital restrictions on bank interest margins is under severe debate [20,21]. examine the OECD and Bangladeshi banking sectors respectively, and find higher regulatory restrictions trend to reduce the interest margin as a result of lower bank profitability, whereas Rahman, Zheng [11] examine the Bangladeshi commercial banking and find higher regulatory restrictions trend to induce the interest margin. These inconclusive natures of previous literature raise the question of whether regulatory capital substantially affects bank interest margin.

The aim of the study is to address the impact of capital and default risk on bank interest margins. Recent literature on emerging economies is limited in providing a comparative standing of the cost of financial intermediation before and during the COVID period. Even those authors covered the COVID period, but the separate regression for the during COVID period is unraveled [2,22,23]. Moreover, past studies ignored the moderating effect of default risk on bank interest margin, which is a significant determinant [24,25]. This study further investigates the effect of all the major events in the globe and Bangladesh on bank interest margins (GFC, Bangladesh capital market crash, COVID period).

We use a panel dataset of 32 Bangladeshi commercial banks from 2000 to 2022. Using dynamic panel two-step GMM, we find vibrant evidence that higher regulatory capital restriction reduces the bank interest margin. In contrast, higher default risk induces the bank interest margin in Bangladesh. We have executed numerous robustness tests to justify our main findings.

This study extended the existing literature in the tiniest three ways; ***first*,** this study is the pioneer that inspects the impact of regulatory capital with the moderating role of default risk on bank interest margins in Bangladesh and the world.

***Second***, this study is a unique extension of the dealership model [26] by calculating the cost functions through Stochastic Frontier Analysis (SFA), whereas most of the previous studies [25] contribute to the dealership model by adding cost functions manually that is simply the ratio of operating cost to total cost.

***Third*,** this study comprehensively considers the major events that shook the economy. For example, it considers the effect of capital and risk on bank interest margins during the recent global financial crisis, the recent pandemic (COVID-19), and the capital market crash period. Thus, the findings seek the interest of the broader aspects of readers and the policy makers.

We selected Bangladesh as an ideal laboratory to inspect our hypothesis for several reasons. Among them, Bangladesh's banking sector underwent several regulatory reforms in our sample period, such as implementing Basel II in 2007 and partially implementing Basel III in 2014. Moreover, the consistent growth of about 6 % in the post-millennium period represents the emerging economies' central benchmark economy. Regarding bank margin, the COVID period is undiscovered [2], we thus introduced here the pre and during the COVID period in separate regression. Moreover, the post-millennium 23 years of data coverage makes this study more robust in the debate on the bank margin domain. Since the data laboratory is in Bangladesh, the pioneer emerging economy, the external financing needs are highly based on the capital market. Tactlessly, the capital market crash since 2010 still runs with dubiousness. Thus, the separate regression for this tragic fall seeks immediate attention.

Hence, the findings could be widespread in other developing and emerging economies in parallel economic conditions. This study will answer the following questions: *Do regulatory capital and default risk reduce bank interest margins in Bangladesh? Does default risk moderate the relationship between regulatory capital and bank interest margin in Bangladesh?*

The rest of the paper is designed as follows: Section 2 consists of a literature review; research design is presented in Section 3, while empirical findings are shown in Section 4. Finally, the conclusion in section 5 and policy implications are presented in section 6.

## 2 Theoretical background and literature review

### 2.1 Theoretical background

Regarding the impact of capital regulation on bank interest margin, supporters argued that the impact would be negative on bank margin [21]. Addressing the bankruptcy costs theory, the shareholders are not regularly required to make a persistent return on investing in high-capitalized banks. Since well-capitalized banks are operated by staying far from default risk, it would create safer hedging for potential investors and satisfy the lower return on investment. The above logic is straightforward. Increasing the equity capital in the capital structure assists in reducing the possibility of bank default, and the banks seem to be safer. Consequently, the bank interest margins will decrease as the shareholders' risk-based required return will reduce. Moreover, banks normally retain more capital than required capital necessities, assisting in creating capital buffers. Capital buffer theory states that an increase in capital buffer above the ceiling reduces the bank risk [27].

Sometimes, in response to stringent capital requirements, banks are cutting their capital buffers [28], and therefore it would reduce the cost of capital.

Similarly, banks may hold more capital to get a competitive advantage and enjoy the market's good share price [21]. The underlying logic tells us that stringent regulatory capital restrictions negatively influence the banks' interest margins.

Nonetheless, some other issues regarding banking regulation could be addressed. For example, there has been a growing number of shadow banking activities in Bangladesh over time, which are unregulated.^4^ Shadow banks are alternative intermediaries of traditional banking activities, placing competition under increased regulation of the mainstream banking institutes [29].

Stringent capital regulation increases shadow banking activities, and in the presence of such, the efficacy of capital regulation is lost [30]. When traditional banks are ignored as a resort of funds, disintermediation occurs [31]. Disintermediation is increasingly becoming a new phenomenon in developing countries [32].

Regarding higher capital requirements, the critics argue that retaining a higher capital ratio would endanger banks and unfavorably affect the banking production process. Since there is some opportunity cost of holding extra capital, it should be added to the banks' operating cost, which may turn into banking system inefficiency. Moreover, following the corporate finance theory of capital formation, the critic argues that equity is an expensive source of funding, and a portion increase in the capital might increase the overall weighted average cost of capital, which might have a positive relationship with bank interest margin [11]. For example, IIF [33] represents more than four hundred financial institutions worldwide and finds that in the USA, the cost of credit would be 5 percentage points higher in response to the Basel III implementation. Likewise, Wong, Fong [34] studied the Hong Kong banking sector and found that a 1 % increase in capital would reduce productivity by 4.2 base points in the long run. Similarly, Naceur and Kandil [24] argue that an increase in regulatory capital requires banks to transform deposit financing by reducing shareholder surplus. This cutting shareholder's surplus enhances the likelihood of loss, and thus it would increase the cost of bank service.

Regarding the impact of default risk on the bank interest margin, we argue that well-capitalized banks (big banks) operate in the safe zone by staying far from default risk [35], which could heterogeneously reduce the cost of bank credit. Higher possibilities of default risk show endanger the bank's capacity to make lending and deteriorate economic productivity. As a result, the bank's overhead cost will increase; accordingly, the bank's clearance of this extra cost of production to the obligors would translate into a higher bank interest margin.

Alternatively, the problem of bank default may not always relate to lending failure. Sometimes, banks may tend to show their default risk to enjoy some facilities such as taxation OTC (Over the Counter Market) trading facility, creating a market strategy. In this situation, bank default may not subprime the bank interest margin. Similarly, during the bad period, banks might take some indeed overcoming strategy from default. Consequently, banks should emphasize the right financing by creating some attractive facilities for borrowers. If so, banks may create an overcoming strategy to reduce their bank margin in the short run. Thus, the impact of default risk on bank interest margins is an undiscovered policy dialogue that deserves particular attention in this research domain.

### 2.2 Regulatory Capital and the bank interest margin

The stability of the banking industry is an indication of any economy's capacity to withstand shocks [36]. Similarly, factors like financial inclusion lead to higher banking sector stability [37]. However, weak institutional settings adversely affect firm-level decisions [38]. Ensuring better country-level governance is linked with increased capital ratios, which could enhance banking stability [39].

Banking intermediary runs through collecting funds (Deposit) and distributing those funds to the productive sector (Lending). Initially, the banks need a base of the fund (Capital) to enter the intermediary process. Thus, capital and interest margin (Lending- Deposit) are the two key determinates of banking research. Essentially, Zheng, Rahman [21] examine the 32 Bangladeshi commercial banks over the post-millennium 16-year period of data and find that capital regulation has an adverse and statistically significant relationship with the bank's cost of financial intermediation (interest margin). Moreover, they witnessed that the banking system's inefficiency and higher financial intermediation (total loan over total deposit) positively affected the bank margins. Besides, management efficiency and income diversification are negatively and significantly associated with bank interest margins. Following Zheng, Rahman [21], this study produces complementary findings, creating new dimensions of incorporating default risk in the model.

Naceur and Kandil [24] studied the Egyptian banking sector from 1989 to 2004 and found that stringent capital requirements positively correlate with the bank's intermediation cost. Moreover, they employed some bank-level variables like management efficiency and reserves to assist in inducing intermediation costs. However, cost inefficiency (the total operating cost to total assets) and market power are adversely associated with banks' cost of intermediation.

Moreover, Ashraf [40] examines the 37 emerging economies from 1998 to 2012, focusing on the impact of trade and capital openness on bank merging. They find that more international openness of trade and capital flow assists in formulating a more effective banking sector liberation policy, which would reduce the cost of bank credit.

However, the banking deregulation policy became a policy dialogue in the aftermath debate of the recent world financial crisis of 2008. In this context, the Basel Committee on Banking Supervision (BCBS) delivered the Basel III accord mechanism to capture the future likelihood of crisis momentum. In what follows, Miles, Yang [41] conclude that the change in capital affects banks' economic output, and the ultimate consequence turns to the bank's interest margin.

Following Ho and Saunders [26] dealership model, several scholars tested the impact of capital ratio (owners' equity to total assets) on bank interest margins among them [25,42,43] find the positive and significant association with bank interest margin. However [44,45], find a negative and significant association with bank interest margin. Besides, Gounder and Sharma [46] find the impact of capital ratio on bank interest margin is insignificant.

The excessive capital requirement puts pressure on bank shareholders and managers to keep the required capital intact; to make that prescribed reserve money, the bank should increase its cost of lending money. As a consequence, the increased loan cost enhances the bank credit cost. The result is an upsurge in the net interest margin is accompanied by a decline in the bank's earnings [47]. Increasing the bank margin may be due to the inducing of monitoring costs beard by managers who control banks^5^. Thus, measuring the bank's inefficiency banking margin is an essential tool [48,49].

In sum, the effect of regulatory capital regulation on bank interest margins is mostly inconclusive. For instance, Naceur and Kandil [24] find a positive association when examining the Egyptian banking systems. Similarly, Rahman, Zheng [11] also found a positive association, whereas Zheng, Rahman [21] found a negative association by examining the Bangladeshi banking sector. Thus, the study's motivation has been justified by the previous inconclusive nature of the literature. The above research and our succinct thinking led to the development of the following alternative hypothesis.

H1 There is an adverse association between regulatory capital requirement and bank interest margin

### 2.3 Default Risk and the bank interest margin

Next, the effect of default risk on bank interest margin has not produced enough evidence. Most of the previous studies following the dealership model [26] examine the impact of credit risk on bank interest margin; however, this study's relational directions could generate a new gateway for future research. A higher lousy loan from the trifling investment basket created the recent crisis and repercussions debate on liquidity shortage. The larger portion of earning assets on total assets is the prediction of higher risk-taking and liquidity crises. Consequently, the higher possibilities of defaults on loans generate higher bank costs that are ultimately transferred to the borrower. Thus, higher default risk has a substantial positive impact on bank interest margin.

However, emerging economies like Bangladesh face some external difficulties, which might significantly affect banking services. Among them, the higher involvement of political people in bank management has created a significant problem in recent days. An increase in political and economic risks induces a risk-taking attitude in banks across the globe [50]. Political risk significantly influences credit risk [51]. Besides, increasing capital requirements reduces credit risk in emerging economies [52].

With the excessive involvement of politically connected people in loan granting, the bank loan screening process might be severely hampered. As a result, the volume of lousy investment increases unexpectedly. The regulatory authority may not perform their prescribed duties when state decision-makers are involved in the loan screening process. Addressing this condition, several scholars Haque and Brown [53], examine and develop a '*public and private view'* of the hypothesis. Public interest view connected with the adverse association between regulatory capital requirements and bank margin. The public view advises that the supervisory authority can protect against market failures by directly nursing the banks. By doing so, stringent regulation and supervision condense harming integrity in lending, increase capital formation efficiency, assist in intensifying the competition, and lessen bank service costs [54].

On the contrary, the private interest view proposes regulatory authority regulation for the interested groups, not for the general public, to decrease the banking sector's efficiency. Such views also established that rigid regulation and supervision are expected to lead to dropping veracity in lending, which would support persuading bank interest margins [55]. Politicians and state supervisors exploit their paybacks and may not have enticements to defend against market collapse. Rather, they use the regulation for forming individual interest crowds, such as banks themselves, to generate professional self-interest or the politically well-linked, which may advocate government-owned banks. Thus, the adverse banking environment may cause higher default risk and bank margins as well.

Numerous previous studies examine the influence of credit risk on bank interest margins based on the dealership model [25,43,48], and most of them find a positive association with bank interest margin. Since credit risk and default risk are the eventual proxies of risk-taking, we thus set up our hypothesis by following the previous literature on bank interest margin based on the dealership model, and can be seen as follows.

H2 There is a positive association between default risk and bank interest margin.

### 2.4 Bank interest margin, synergy of regulatory Capital and default Risk

As we emphasize the regulatory capital requirements and default risk impact on bank interest margin, we next develop a theoretical background based on the '*regulatory and moral hazard hypothesis*.' We argue when a bank suffers a high default probability, it may react negatively to the stringent restrictions on banking activities, which would have a positive impact on the bank's interest margin. Thus, we suggest the regulatory authority to ease the capital restrictions, especially for default banks. That might positively influence survival and come back to a solvent bank. The *'regulatory hypothesis'* suggests that stringent capital regulation helps banks lower risk regarding loan screening and processing. Strict capital regulation helps to reduce the probability of default; consequently, it could make a path for reducing non-performing loans. If the non-performing loan is reduced, the banks might not need to charge a higher interest margin. Based on the regulatory hypothesis, we may assume regulatory capital and default loan interactions have an adverse effect on bank interest margins. However, the statutory authorities should especially care about the default banks to overcome the situation that will bring well-being to the banking sector. The *moral hazard hypothesis suggests* that the lower capitalized banks face suffering as inferiority complexity compared to well-capitalized banks. Thus, they would try to take a high risk in loan processing. If so, the probability of default will be high, and as a consequence, the bank interest margin will be high. Based on the moral hazard problem, the regulatory authority should assist small banks in reducing their probability of default, which would consequently reduce the banks' interest margins.

Similarly, examining Pakistani commercial banks [56] finds higher risk aversion through increasing regulatory capital could reduce the credit risk by charging a lower bid price. While Gulati, Goswami [57] study of Indian banking found that charging a lower margin could reduce the banks' profitability, which would assist in increasing the probability of default. Hence, we assume that the interaction of regulatory restrictions and default risk could reduce the net interest margin in the long run.

H3 The interactions of regulatory capital restrictions and default risk have a negative impact on bank interest margin.

## 3 Research design

### 3.1 Sample and time Frame

**Fig. 2:**
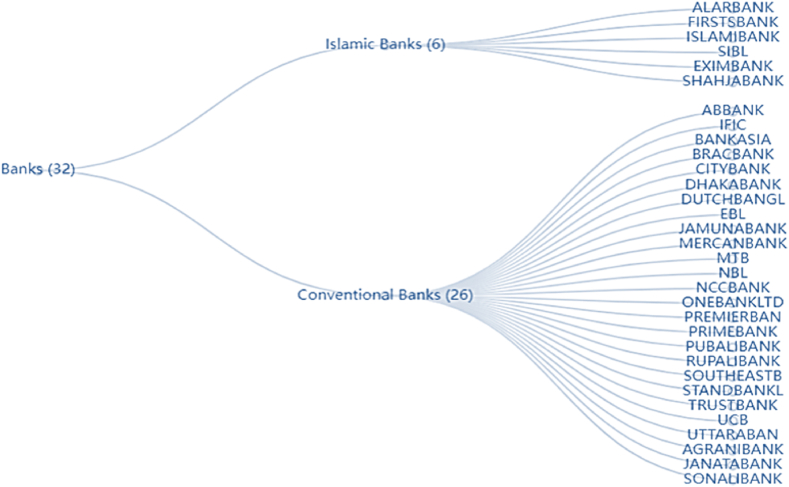
Tree graph of the banks under study using ticker symbols.

The data castoff in this study collected from several sources; bank-level variables data are collected from the BankFocus database compiled by the Bureau Van Dijk, focusing on the consolidated financial statement and audited annual report from the period of 2000–2022 of each bank. We have conducted data wrangling to make data useable. Data construction had been started by erasing a lot of missing figures. We need to delete all negative and zero values for calculating SFA of cost inefficiency, as all input and output variables, including dependent variables, need to convert to natural logarithm form. However, the BankFocus database regarding Bangladesh is full of missing figures; we have decided to manually fill in the data from the audited annual report. In that case, we find the audited annual report and BankFocus data presentation systems are not unique. For instance, in the case of the total assets, the BankFocus database presented after deducting non-performing loans, but the audited annual report presented the accumulated amount, including the non-performing loan. Thus, we follow the audited annual report to construct our dataset. After all, the final dataset consists of 598 observations of 32 commercial banks for 2000–2022. The list of the banks is illustrated in Fig. 2.

Data for Capital Stringency (CAPR) and Entry Restrictions (ENRES) were gathered from Ref. [10]. Data for the KKZ index collected from the Worldwide Governance Indicators (WGI) database of [58]. We collect data for macroeconomic variables from the World Development Indicators (WDI) of the World Bank. Finally, data for macroeconomic variables are attached to bank-level control and industry-specific variables to complete the final dataset. Table 1 below is a detailed explanation of the data source that we used in this study.

**Table 1:** Description of the variables.

Variables	Symbol	Descriptions	Sources of variable
***Dependent Variables***			
Bank Interest Margin	NIM1	Equals the ratio of net interest revenue over total earning assets of every bank	[59]
	NIM2	The ratio of net interest revenue over total assets of each bank	[21]
***Main Independent Variables***			
Regulatory Capital	CAR	Equals the ratio of owner's equity to total assets. i.e., capital adequacy ratio	[21]
	CAPD	Dummy variable calculated as the 1 for the period of Basel III implementation and after that, 0 otherwise	Author's Idea
	CAPR^6^	Capital stringency is measured based on 10 questions proposed by the World Bank. The index varies from 0 to 10, where higher values represent higher regulatory restrictions and vice versa.	[10]
	ENRES^7^	Entry or legal restrictions measured based on 8 questions proposed by the World Bank. The index ranges from 0 to 8, where greater values represent higher entry restrictions in the banking business and vice versa.	[10]
Default Risk	ZSCORE	Equals −1*[log [(ROA + CAR)/σ(ROA)], where ROA and CAR are the yearly return on assets before taxes and shareholders' fund to total assets ratios, respectively. σ(ROA) is the standard deviation of yearly return on total assets before taxes calculated over each bank sample period rolling window. A greater value of ZSCORE denotes higher bank default risk, and vice versa.	[60]
Regcap* Default Risk	REGDR	The interaction term of Capital Adequacy Ratio (CAR) and Default Risk (ZSCORE)	Author's Idea
***Bank-independent control variables***			
Cost Inefficiency	COSTINEFF	Estimated using stochastic frontier 4.1	Authors' calculations
Operating Cost Ratio	OPERRO	Calculated as the portion of operating cost to total assets of each bank	[43]
Management Efficiency	MANEFF	Equals the portion of interest-bearing assets to total assets of each bank	[11]
Reserves	RSVS	Equals the ratio of annual reserve in the central bank to total assets of every bank	[25]
Funding Strength	TLTD	Calculated as the ratio of total loan to total deposit of each bank	[11]
Income Diversification	INDI	Calculated as the ratio of non-interest revenue over total operating revenue of the individual bank	[11]
***Industry-Specific Variable***			
Market Concentration	CR3	Equals the ratio of the three largest bank assets to total banking assets	[11]
	HHI	Hirschman-Herfindahl Index calculated as the sum of the square of the market share of every bank each year	[25]
***Macroeconomic Variable***			
Governance Index	KKZ	Composite the country score in the area of govt. effectiveness, political stability, voice and accountability, the rule of law, regulatory quality, and control of corruption. The index varies from −15 to 15, with higher values representing more governance practices in the country	[45,58]
Interest Rates Risk	SDMIR	Calculated as the yearly standard deviation of the once-a-month average of the daily money market interest rates.	Inter. Financial Statistics (IMF)
Inflation, Consumer Prices (annual %)	INF	The annual rate of inflation (%)	WDI of WB^8^
***Other variables***			
Crisis Period Analysis	CRISIS	Quantify as the value of 1 from 2007 to 2009 and 0 otherwise	[45]
Capital Market Crash Analysis	CRASH	Quantify as the value of 1 for the period of 2010–2011 and 0 otherwise	Authors' Idea

### 3.2 Variable explanations

#### 3.2.1 Dependent variables

Following recent literature [21,[23], [24], [25]], we captured bank interest margin by two proxies, NIM1 and NIM2. NIM1 is calculated as the portion of net interest revenue (interest revenue minus interest expenses) over total interest-bearing assets, and NIM2 is measured as net interest revenue (interest revenue minus interest expenses) to each bank's total assets. A higher value of NIM1 and NIM2 represents a higher bank interest margin and vice versa. In the empirical analysis, NIM1 is considered for the main results, whereas NIM2 is used for robustness checks.

#### 3.2.2 Main variables

Since we emphasize capital and risk on bank interest margin, these two variables are essentially our variables of interest. Following the previous literature [11,21,24,45,61], we captured regulatory capital by five proxies such as CAR, CAPD, CAPR, and ENRES. Regarding default risk, we captured by ZSCORE, which is widely used by Refs. [62,63], where the higher value of ZSCORE represents higher default risk and vice versa.

The capital adequacy ratio (CAR) is calculated as the proportion of owners' equity to each bank's total assets. Higher ratios represent the banks that are well-capitalized, indicating the bank is operated with a risk aversion policy [45]. The relationship of CAR with bank interest margin is under severe debate. For instance, higher capitalized banks are relatively safe depositors and potential investors, which might reduce the cost of credit. On the contrary, higher capital in the capital structure represents greater risk aversion, which may motivate banks to invest in less risky projects with a lower return, resulting in a lower bank interest margin [45]. Likewise, Demirgüç-Kunt, Laeven [64] argue that well-capitalized banks are less likely to default, which could negatively impact the bank interest margin.

The capital dummy (CAPD) indicates switching from Basel II to Basel III on bank interest margins in the Bangladeshi banking sector. We quantify this variable as equal 1 following the period of Basel III (2014) and, after that, 0 otherwise. Switching from Basel II to Basel III in full phase was the major regulatory reform in the Bangladeshi banking sector. Thus, the impact on the bank margin of the major regulatory reforms should have a special concentration on the regulatory authorities and policymakers.

The capital stringency (CAPR) index consists of 10 questions regarding the injection of new capital into the existing capital structure of the bank proposed by Ref. [10]. A higher index represents a higher stringency of regulatory capital requirements and vice versa.

Entry Restrictions (ENRES) index consists of 8 questions regarding the restrictions on entering the banking business developed by the World Bank in the Bank Regulation and Supervision Database. A higher index represents greater restrictiveness on entering the banking business. Poghosyan [45] examines this variable as the fraction of entities denied and finds its positive association with bank interest margin in LICs and EMs. Arguing that restricting the entering banking service of the incumbent banks allows existing banks to charge higher-margin since they may enjoy higher bargaining power with potential borrowers regarding the cost of bank credit. However, we argue alternative views of legal restrictions that greater restrictions on entering banks allow regulatory authorities to make discipline through supervisory power and private monitoring. The banks tend to charge with lower interest margins.

Later, we scrutinize the effect of the interaction of regulatory capital and default risk on the bank interest margin. We generate a variable by interacting between regulatory capital (CAR) and default risk (ZSCORE). Based on the theoretical background, we develop an argument that regulatory authorities should be liberal in the restrictions on capital for the time being to overcome the probability of default, and we named this period a nursing period.

#### 3.2.3 Control variables

This study uses bank-specific, industry-specific, and macroeconomic-level controls. Six bank-specific variables are used in the study to control for any bank-level impact. Cost Inefficiency (COSTINEFF) is estimated using the SFA software Frontier 4.1. The cost function of the banks is estimated, and the factors contributing to the cost inefficiency are identified [65,66].

Operating Cost Ratio (OPERRO) is measured as the ratio of operating cost to total assets of individual banks [43]. This ratio measures the operational efficiency of banks. Another measure of efficiency is Management Efficiency (MANEFF), which is the ratio between interest-bearing assets and total assets of each particular bank [11]. Other bank-specific variables are Reserves (RSVS), Funding Strength (TLTD), and Income Diversification (INDI). RSVS is the ratio between reserves in the central bank (i.e., Bangladesh bank) annually to total bank assets [25]. The RSVS ratio is considered significant since the financial crisis and constitutes a significant portion of the bank assets [67].

TLTD is measured as the ratio between total loan to total deposit (Funding Strength) for each individual bank [11,21], which they named as financial intermediation. TLTD measures liquidity risk as volatile liquidity can adversely impact baking activities [68].

Finally, the INDI is calculated as non-interest revenue divided by the total operating revenue of each bank [11]. INDI measures the diversification of revenues by the banks and is a major indicator of bank performance.

For industry-level control variables, Market Concentration (CR3 and HHI) is considered [11,21,25]. Market Concentration measures the market competitiveness of the individual banks. Governance Index (KKZ), Interest Rates Risk (SDMIR), inflation, and consumer prices annual percentage (INF) are used to account for macroeconomic factors. Two dummy variables, Crisis Period Analysis (CRISIS) and Capital Market Crash Analysis (CRASH), are used to control for time-specific impact. Detailed operational definitions of these variables can be found in Table 1.

### 3.3 Experiential methodology

This research applied the two-step system GMM^9^ Estimation was, followed by Refs. [69,70] to examine the impact of capital and risk on bank interest margin. Due to a vibrant model's dynamic nature could not be estimated with the standard OLS technique, as it might convey unreliable results for the intense correlation between fixed effect and lagged dependent variables [71]. The fixed effect regression estimation may also be unreliable because of the unobserved dynamic interlink within the variables [72]. Further, the fixed and random effect estimation, standard OLS model, cannot consider potential heteroskedasticity, endogeneity, and serial correlations in the models. As a result, this research applies the dynamic panel system GMM estimation method to account for potential heteroskedasticity, endogeneity, and serial correlation problems among the variables [73]. In the GMM estimator case, we notice two variations are available: differenced and system GMM method. The difference GMM estimator originated by Ref. [74] only deliberate the results largely based on the serial correlation of lagged levels of X_i,t-1_ as an instrument in the equation traced as first-differencing. However, the system GMM methodology originated by Refs. [69,70] endorses the system of first-differenced and level equations, where lags level and lags of first-difference is also used as an instrument. This system GMM estimator is an established and high-power estimation technique over differenced GMM. Furthermore, following the guideline of [75], we accomplish finite-sample rectification to adjust the standard errors using the two-step estimation for a given bank over the sample period.

Moreover, this study used the two-step GMM system for estimation, which accounts for concern over unobserved heterogeneity, autocorrelation, and endogeneity [76]. Most of the studies in the field also applied the two-step system GMM for similar reasons [21,23,24]. The data Non-Stationary test are presented in the Appendix Tablet A1.

### 3.4 Specified model

**Fig. 3:**
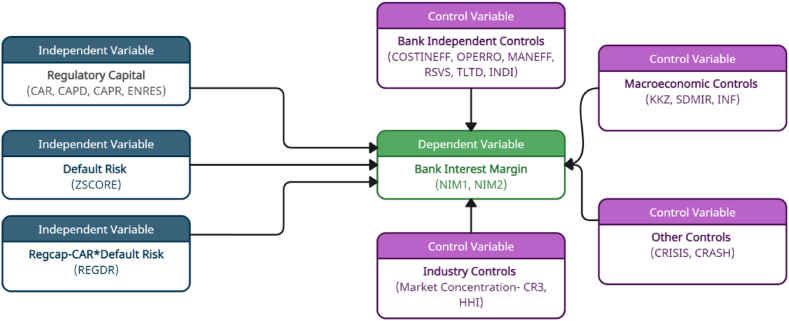
Conceptual model of the study.

To measure the impact of capital and risk on bank interest margin, we follow [43] single-stage modified dealership model. With the variable of interest, we also consider some other important determinants. After all, we develop the following linear empirical model 1.Based on the different proxies of regulatory capital; we develop the model (2–5). The birds-eye view of the model can be found in Fig. 3.

$${\text{NIM}}_{i,t}=\beta_{0}+\beta_{1}{\text{NIM}}_{i,t‐1}+\beta_{2}{\text{Regulatory}\;\text{Capital}}_{i,t}+\beta_{3}{\text{Default}\;\text{Risk}}_{i,t}+\beta_{4}{\left(\text{Regcap}\;x\;\text{Default}\;\text{Risk}\right)}_{i,t}$$

(1) $$+\sum_{b=1}^{B}\lambda_{b}Y_{\text{it}}^{b}+\beta_{5}{\text{Market}\;\text{Concentration}}_{i,t}+\sum_{m=1}^{M}\delta_{m}Y_{\text{it}}^{m}+{\text{TD}}_{t}+\varepsilon_{\text{it}}$$

Where the subscripts i and t denote bank and time, respectively. Net interest margin (NIM) is a proxy of bank interest margin. $\beta_{0}$ is a constant term. $Y_{\text{it}}$ with superscripts *b,* and *m* are the vectors of bank and macroeconomic-specific variables, respectively, and $\in_{\text{it}}$ is the error term. The bank-specific variables are cost efficiency, management efficiency, reserves, funding strength, and income diversification. The macroeconomic variables are the governance index, interest rate risk, and inflation. Bank-specific heterogeneity is neutralized by the fixed effects intercept term C, and the time horizon variation is seized by employing the vector of time dummies TD. The detailed definitions and references of variables are presented in Table 1. The below figure-3 represents the conceptual model of the study

After considering the alternative proxies of regulatory capital, the revised models will be the following.

Model 2 with the CAR: Here CAR equals the ratio of owner's equity to total assets

$${\text{NIM}}_{i,t}=\beta_{0}+\beta_{1}{\text{NIM}}_{i,t‐1}+\beta_{2}{\text{CAR}}_{i,t}+\beta_{3}{\text{Default}\;\text{Risk}}_{i,t}+\beta_{4}{\left(\text{Regcap}*\;\text{Default}\;\text{Risk}\right)}_{i,t}$$

(2) $$+\sum_{b=1}^{B}\lambda_{b}Y_{\text{it}}^{b}+\beta_{5}{\text{Market}\;\text{Concentration}}_{i,t}+\sum_{m=1}^{M}\delta_{m}Y_{\text{it}}^{m}+{\text{TD}}_{t}+\varepsilon_{\text{it}}$$

Model 3 with the CAPD: Here, CAPD is a dummy variable equals 1 for the period of application of Basel III and after that, 0 otherwise

$${\text{NIM}}_{i,t}=\beta_{0}+\beta_{1}{\text{NIM}}_{i,t‐1}+\beta_{2}{\text{CAPD}}_{i,t}+\beta_{3}{\text{Default}\;\text{Risk}}_{i,t}+\beta_{4}{\left(\text{Regcap}*\;\text{Default}\;\text{Risk}\right)}_{i,t}$$

(3) $$+\sum_{b=1}^{B}\lambda_{b}Y_{\text{it}}^{b}+\beta_{5}{\text{Market}\;\text{Concentration}}_{i,t}+\sum_{m=1}^{M}\delta_{m}Y_{\text{it}}^{m}+{\text{TD}}_{t}+\varepsilon_{\text{it}}$$

Model 4 with the CAPR: Capital stringency index calculated based on 10 questions suggested by [10].

$${\text{NIM}}_{i,t}=\beta_{0}+\beta_{1}{\text{NIM}}_{i,t‐1}+\beta_{2}{\text{CAPR}}_{i,t}+\beta_{3}{\text{Default}\;\text{Risk}}_{i,t}+\beta_{4}{\left(\text{Regcap}*\;\text{Default}\;\text{Risk}\right)}_{i,t}$$

(4) $$+\sum_{b=1}^{B}\lambda_{b}Y_{\text{it}}^{b}+\beta_{5}{\text{Market}\;\text{Concentration}}_{i,t}+\sum_{m=1}^{M}\delta_{m}Y_{\text{it}}^{m}+{\text{TD}}_{t}+\varepsilon_{\text{it}}$$

Model 5 with the ENRES: Entry restrictions index calculated based on 8 questions suggested by [10].

$${\text{NIM}}_{i,t}=\beta_{0}+\beta_{1}{\text{NIM}}_{i,t‐1}+\beta_{2}{\text{ENRES}}_{i,t}+\beta_{3}{\text{Default}\;\text{Risk}}_{i,t}+\beta_{4}{\left(\text{Regcap}*\;\text{Default}\;\text{Risk}\right)}_{i,t}$$

(5) $$+\sum_{b=1}^{B}\lambda_{b}Y_{\text{it}}^{b}+\beta_{5}{\text{Market}\;\text{Concentration}}_{i,t}+\sum_{m=1}^{M}\delta_{m}Y_{\text{it}}^{m}+{\text{TD}}_{t}+\varepsilon_{\text{it}}$$

For calculating cost inefficiency, we use SFA to develop the following model 6.

$${\text{lnTC}}_{\text{it}}=\beta_{0}+\sum_{n=1}^{3}\beta_{n}{\ln\;P}_{\text{nit}}+\sum_{k=1}^{2}\delta_{k}{\text{lnY}}_{\text{kit}}+\sum_{n=1}^{3}\sum_{m=1}^{3}\beta_{\text{nm}}{\ln\;P_{\text{nit}}\text{lnP}}_{\text{mit}}+\sum_{k=1}^{2}\sum_{j=1}^{2}\delta_{\text{kj}}{\ln\;Y_{\text{kit}}\text{lnY}}_{\text{jit}}$$

(6) $$+\sum_{n=1}^{3}\sum_{k=1}^{2}\gamma_{\text{nk}}{\ln\;P_{\text{nit}}\text{lnY}}_{\text{kit}}+\in_{\text{it}}$$

Here in model 6, the dependent variable is total cost (TC), which is defined as the sum of total interest and operating expenses. In the specification of the inputs and outputs, we follow the intermediation approach and specify input prices (*p*) as the price of labor (PL), the price of fixed asset (PF), and the price of funds (PF)^10^. The outputs (*Y*) are defined as total loans (TN) and other earning assets (OEA) [77].

## 4 Empirical findings

**Fig. 4:**
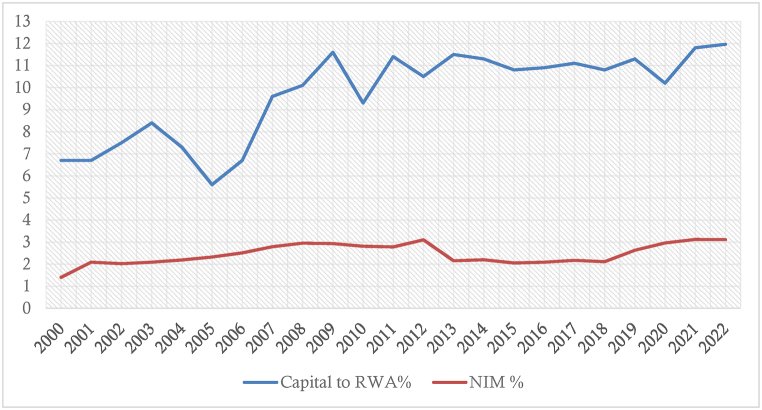
Yearly (Average) capital to risk-weighted assets and net interest margin of all sample banks (Data source: Annual reports).

Fig. 4 visualizes a comparative position of NIM and capital to risk-weighted assets. From an overall perspective, we can already observe some insights that require interpretations. The growth of NIM remained quite stable over time, whereas the capital to risk-weighted assets was volatile. The rate of capital to risk-weighted assets increased over time and was the lowest during 2005. On the other hand, the highest NIM was achieved during 2012, which was short-lived and fell gradually from the subsequent years. During the global financial crisis (2007–2009), bank interest margins increased. Similarly, the capital to risk-weighted assets also increased during the crisis period. These findings motivate us to further test the relationships and provide an empirical overview of the situation.

### 4.1 Descriptive statistics and correlation Matrix

Table 2 represents the descriptive statistics of the variables employed in the models. The highest standard deviation of the employed variable is INF-2.21, implying that the data outliers are not a significant taunt in the models.

**Table-2:** Summary statistics of the variables.

Variables		Obs	Mean	Maximum	Minimum	Std. deviation
*Dependent variables*						
Bank Interest Margin (NIM1)		598	0.02	0.05	−0.01	0.01
*Main independent variables*						
Regulatory Capital	CAR	598	0.12	1.66	−0.01	0.09
	CAPD	598	0.62	1.00	0.00	0.49
	CAPR	598	4.31	6.00	3.00	1.30
	ENRES	598	6.35	7.00	6.00	0.48
Default Risk (ZSCORE)		598	598	1.50	−3.52	0.80
Regcap* Default Risk (REGDR)		598	598	2.49	0.036	0.072
*Bank-level control variables*						
COSTINEFF		598	0.13	0.83	0.01	0.12
MANEFF		598	0.87	0.99	0.66	0.05
RSVS		598	0.06	0.19	0.00	0.02
TLTD		598	0.81	1.54	0.01	0.12
INDI		598	0.58	1.72	0.00	0.20
*Industry-level control variable*						
CR3		598	0.17	0.28	0.12	0.04
*Macroeconomic variables*						
KKZ		598	−5.37	−4.40	−6.71	0.60
SDMIR		598	0.03	0.08	0.01	0.02
INF		598	6.71	10.70	2.01	2.21

Further, Table 3 represents Pearson's correlation coefficient among the variables. The highest correlation between CAPD and CAPR is 0.70^11^, which indicate the problem of multicollinearity does not pose any challenge to us. Moreover, this study also presents the result of VIF (Variance Inflation Factor) test for all the related variables. In all cases, the VIF score is below 10, which is within the cutoff value. Hence, the issue of multicollinearity is nonexistent [78].

**Table 3:** Correlation among the variables.

Variables	NIM1	ZSCORE	CAR	CAPD	CAPR	ENRES	REGDR	COSTINEFF	MANEFF	RSVS	TLTD	INDI	CR3	KKZ	SDMIR	INF	VIF
NIM1	1																**1.05**
ZSCORE	0.53*	1															**1.27**
CAR	−0.13	−0.18	1														**1.04**
CAPD	−0.11	−0.27*	0.07	1													**1.14**
CAPR	−0.02	−0.21	0.07	0.7	1												**1.09**
ENRES	−0.02	−0.15	0.06*	0.57	0.65	1											**1.08**
REGDR	−0.13	−0.45*	0.31	0.28*	0.61	0.06	1										**1.01**
COSTINEFF	−0.15*	−0.05	0.02	0.58	0.64*	0.61	0.09*	1									**1.11**
MANEFF	−0.01	−0.27*	0	−0.25	−0.24	−0.2	0.04*	−0.25	1								**1.07**
RSVS	0.26	−0.24	0.03	0.21*	0.2	0.17	0.12	0.13	−0.46*	1							**1.24**
TLTD	0.40*	−0.42	0.04	0.17	0.08	0.02	−0.36	−0.09	0.26	0.14*	1						**1.09**
INDI	−0.73	0.43*	−0.06	−0.04	−0.05	−0.05	0.42*	0.04	−0.04	−0.31	−0.31	1					**1.03**
CR3	−0.03	0.22*	0.01	−0.55	−0.54	−0.62*	−0.19	−0.62	0.18	−0.22	−0.09	0.07*	1				**1.08**
KKZ	−0.03	−0.02	0.07	0.4	0.41	0.35	0.03	0.3	−0.14	0.04	−0.07	0.05	−0.14	1			**1.17**
SDMIR	0.16*	−0.06	−0.06	−0.1	−0.31	−0.37	−0.1	−0.2	0.01	0.03	0.13*	−0.03	−0.07	−0.14*	1		**1.19**
INF	0.17*	−0.24	−0.01	0.56	0.41	0.27	0.06	0.22	−0.13	0.13	0.24*	−0.06	−0.38*	−0.29	0	1	**1.04**

Note: The symbols * indicates statistical significance at the 1 % levels.

**Table 4:** Summary of the hypothesis testing.

Hypothesis	Relationships	Expected Sign	Outcome	Comments
H_1_	*Regulatory capital requirement****→****Bank Interest Margin*	(−)	(−)	*Supported*
H_2_	*Default risk****→****Bank Interest Margin*	(+)	(+)	*Supported*
H_3_	Regulatory capital requirement*Default risk***→****Bank Interest Margin*	(−)	(−)	*Supported*

The four-alternative measure of regulatory capital correlation is not 1; justify the use as an alternative measure of capital. In a single-way direction, the correlation coefficient of our main variables proves our final regression estimation using the two-step GMM system. For instance, ZSCORE enters positively with NIM, indicating that there is a positive association between them. Similarly, the negative coefficient of CAR, CAPD, CAPR, and ENRES with NIM indicates a negative association between regulatory capital proxies and bank interest margin. Thus, primarily, we got some relational ideas about our variable of interest, such as regulatory capital, default risk, and bank interest margin. The coefficients of other variables also vary across the sample period.

### 4.2 The effect of Capital and Risk on the bank interest margin: main results

The reported results in Table 5 show the impact of capital and risk on bank interest margin using the two-step system GMM estimation based on Eqs. (2), (3), (4), (5). Here, NIM is the dependent variable that we tested by employing some other independent variables, including capital and risk. As shown, the lagged dependent variable is positively and significantly associated with the bank interest margin, indicating that the impact of the previous year's net interest margin still exists [21].

**Table 5:** The impact of regulatory capital and default risk on the bank interest margin: Main results.

Variables	(1)	(2)	(3)	(4)
	NIM1	NIM1	NIM1	NIM1
NIM1(-1)	0.186*(0.101)	0.190*(0.104)	0.247**(0.102)	0.242**(0.110)
**CAR**	−0.002**(0.001)			
**CAPD**		**−0.001(0.001)**		
**CAPR**			−0.001**(0.0006)	
**ENRES**				−0.001*(0.001)
**ZSCORE**	0.002***(0**.0001)**	0.002***(0.001)	0.002***(0.005)	0.002***(0.004)
**REGDR**	−0.001**(0.0005)	−0.002**(0.0009)	−0.001*(0.001)	−0.001**(0.0006)
COSTINEFF	0.241***(0.037)	0.241***(0.039)	0.222***(0.033)	0.223***(0.035)
MANEFF	−0.019**(0.008)	−0.020**(0.008)	−0.019**(0.008)	−0.018**(0.007)
RSVS	−0.009(0.012)	−0.009(0.012)	−0.009(0.013)	−0.009(0.013)
TLTD	0.010***(0.002)	0.011***(0.003)	0.009***(0.002)	0.009***(0.002)
INDI	−0.024***(0.004)	−0.024***(0.004)	−0.023***(0.003)	−0.023***(0.003)
CR3	0.032***(0.009)	0.030***(0.007)	0.017**(0.007)	0.019**(0.009)
KKZ	−0.005**(0.002)	−0.008***(0.003)	−0.007***(0.002)	−0.005***(0.002)
SDMIR	0.037***(0.011)	0.029**(0.012)	0.021(0.016)	0.027**(0.012)
INF	0.0003***(0.001)	0.0004***(0.001)	0.0004***(0.001)	0.0004***(0.001)
CONSTANT	0.021***(0.008)	0.023***(0.009)	0.027***(0.009)	0.031***(0.010)
Sargan test	19.38(0.69)	17.05(0.83)	16.67(0.93)	16.91(0.89)
AR(1)	−3.56(0.00)	−3.51(0.00)	−3.12(0.00)	−3.16(0.00)
AR(2)	−0.30(0.76)	−0.28(0.78)	−0.25(0.80)	−0.33(0.74)
Instruments	21	21	21	21
Observations	598	598	598	598
No. of banks	32	32	32	32
***Analytical tests***				
Hausman endogeneity test (P-value)	34.53***(0.00)	34.66***(0.00)	34.92***(0.00)	35.12***(0.00)
LM serial correlation test (P-value)	151.99***(0.00)	156.34***(0.00)	167.90***(0.00)	169.41***(0.00)
White heteroskedasticity test (P-value)	450.93***(0.00)	312.56***(0.00)	322.91***(0.00)	325.63***(0.00)
Hausman Fixed/Random effect test (P-value)	1.00	1.00	1.00	1.00

Note: The reported results in this table are the impact of capital and risk on bank interest margins. The dependent variable is NIM1 across all models. A higher value of NIM1 denotes a higher bank margin, and vice versa. The estimation technique is the Two-Step System GMM panel regression. 1 %, 5 % and 10 % levels significance represents by ***, ** and *, respectively. Heteroskedasticity-robust standard errors are in parentheses. The alternative hypothesis of the Sargan test is that the instruments used in the models are interrelated with residuals (over-identifying restrictions). Arellano–Bond order AR1 (2) are tests for first (second) order correlation, asymptotically N (0,1). In the system GMM estimation, these tests represent the first-differenced residuals of the estimation. The alternative hypothesis of the Hausman endogeneity test, LM serial correlation test, and White heteroskedasticity test is that the problem of endogeneity, serial correlation, and heteroskedasticity remain in the model. We accept the alternative hypothesis in all models. The Hausman Fixed/Random effect test's alternative hypothesis is that there are fixed effects that exist in the models, and we reject the alternative hypothesis of all models. Details about the variables are presented in Table 1.

CAR enters negative and significant with bank interest margin indicating that higher capitalized banks are enjoying a competitive advantage in the diversified investment opportunity. Consequently, they could reduce the gap between 'ask' and 'bid' prices. Similar results were shown by Refs. [21,44,45]. However, contrary to the findings of [11,25,42,48].

CAPD enters negative but insignificant with bank interest margin, implying that converting from Basel II to Basel III has an adverse effect on bank interest margin; however, the impact is not mentionable. Similar findings also produced by Refs. [11,21]. However, they tested the transition period of Basel I to Basel II.

CAPR comes negative and statistically significant with bank interest margin indicating that the stringent capital requirements assist a bank in charging lower interest on the bank loan.

ENRES enters negative and statistically significant with bank interest margin. It suggests that higher restrictions on entering the banking business assist the regulatory authority in increasing the supervisory plus private monitoring of banking activities. Thus, it would help to reduce the bank's interest margin. However, contrary to the findings of [45].

Capital and risk interaction shows a negative and significant relationship with the bank interest margin and justifies our baseline hypothesis. We reason that the marginal impact of capital regulation assists in reducing the bank interest margin. Moreover, particular banks deserve special attention from the regulatory authority to overcome bad times during the recession period. A summary of the established hypothesis of the study and its outcome is presented in Table 4.

Among the bank-specific variables, cost inefficiency enters positive and statistically significant with bank interest margin. That implies higher cost inefficiency in the banking intermediation-driven bank to increase overhead cost, and the ultimate effect on bank margin becomes positive. Similar findings also produced by Refs. [21,79].

Management efficiency shows a negative and statistically significant association with bank interest margin, indicating that higher-earning assets to total assets signify banking systems efficiency in fund management. Moreover, a higher amount of earning assets helps the bank enjoy the economy of scale in the operational procedure, which might help reduce the cost of lending; therefore, the bank margin will be reduced [11,21]. But contrary to the findings of [24].

Reserve enters negative with bank interest margin, but the relationship is statistically insignificant, suggesting that higher reserve to total assets is a signal for banking systems stability. Moreover, it also represents the credibility of regulatory authority regarding monitoring the banking operation, which helps to reduce the bank interest margin. Similar findings are also found in Ref. [11], contrary to the results [24].

The total loan to total deposit, named as funding strength, enters positively and significantly with the bank interest margin. A higher portion of the deposit in the form of a loan might increase the default probability. If so, the amount of loan loss provision will increase, and consequently, bank loan costs will increase [21,25].

Income diversification shows a negative and statistically significant association with bank interest margin. When a bank can generate more non-interest income, the bank operates to focus on market-based banking rather than bank-based banking. In that case, the bank might enjoy some freedom in setting lower pricing compared to others. Particularly, it releases pressure on generating interest income, and consequently, bank interest margin will be reduced [21].

Market concentration enters positive and significant with bank interest margin, indicating that the customary Structure–Conduct–Performance (SCP) hypothesis is quite present in the Bangladeshi banking sector. When the banks operate with a large market share, they naturally enjoy some monopoly facility in setting the price. If so, they might face lower competition from a potential incumbent, leading them to charge a higher interest margin on the bank loan. Thus, the bank interest margin will increase with the monopoly power of the large banks. Findings in the line of [26,43,45]. However, contrary to the findings of [25,80,81].

KKZ index comes negative and significant with bank interest margin, explaining that greater state institutional quality helps banks operate in a sound and stable environment. Therefore it could marinate to reduce the bank interest margin [45].

Interest rate risk shows a positive and significant relationship with bank interest margin, indicating that those banks are unlike manufacturing firms to maintain inventory. Banks need to make deposit money to retain in inventory nature and need to adjust the interest rate fluctuation loss with bank credit cost. Thus, the bank interest margin will induce to adjust to the fluctuation of short-term interest rates. Similar findings appeared in Refs. [26,43,80]. However, opposing the conclusions of [42,82].

Inflation shows a positive and statistically significant relationship with the bank interest margin in Bangladesh. The reasoning is due to the devalued nature of money's purchasing power; banks need to adjust the inflationary adjustment in the long run. If so, banks need to charge more to the borrowers to recover the loss of purchasing power of money, and therefore, the bank interest rates need to be increased. Supporting findings produced by Refs. [48,80,81]. They are equally conflicting with the results of [82].

Finally, the insignificant P-values of the Sargan test indicate that the instruments employed in this study are not associated with the residuals and satisfy the over-identified restrictions of our models. Moreover, Arellano–Bond order 1 (2) is tested for the first (second) order association. It delivers the required findings that the first-order autocorrelation is significant with a large coefficient, and second-order autocorrelation enters insignificantly small coefficient values. The problem of instrument proliferation does not undermine our results since 23 years of sample period with 32 banks consisting of 21 instruments is not too high in this regard.

### 4.3 Robustness check: alternative proxies and inclusion of time dummies instead of macroeconomic variables

We check the sensitivity of our main findings by employing NIM2 as an alternative proxy of NIM1, operating cost ratio (OPERRO) instead of stochastic cost inefficiency (COSTINEFF) and Hirschman-Herfindahl Index (HHI) instead of CR3 as the proxy of market concentration. Moreover, we replaced the year fixed effect dummies instead of inflation and interest rates risk. NIM2 is calculated as the net interest revenue to each bank's total assets, and the operating cost ratio is calculated as each bank's operating cost to total assets. HHI is calculated as the summation of the square of the market share of the individual bank each year. After all the variables in our estimated Eqs. (2), (3), (4), (5) are replaced, we re-estimate our models using the two-step GMM technique, and the results are reported in Table 6.

**Table 6:** Sensitivity check: Alternative proxy of the interest margin (NIM2), cost inefficiency (OPERRO) and marker concentration (HHI), along with the inclusion of time dummies instead of macroeconomic variables (INF, SDMIR).

Variables	(1)	(2)	(3)	(4)
	NIM2	NIM2	NIM2	NIM2
NIM2(-1)	0.199*(0.103)	0.206*(0.123)	0.258**(0.107)	0.254**(0.112)
**CAR**	−0.002**(0.0006)			
**CAPD**		**−0.004(0.001)**		
**CAPR**			−0.001**(0.004)	
**ENRES**				−0.005**(0**.0003)**
**ZSCORE**	0.002***(0.0005)	0.002***(0.001)	0.002***(0**.0004)**	0.002***(0.0004)
**REGDR**	−0.001**(0.0006)	−0.002**(0.008)	−0.001*(0.001)	−0.001**(0.0005)
OPERRO	0.192***(0.032)	0.191***(0.033)	0.176***(0.028)	0.176***(0.030)
MANEFF	−0.007(0.006)	−0.008(0.006)	−0.008(0.006)	−0.007(0.006)
RSVS	−0.011(0.010)	−0.010(0.010)	−0.011(0.010)	−0.011(0.010)
TLTD	0.009***(0.002)	0.009***(0.003)	0.008***(0.002)	0.007***(0.002)
INDI	−0.020***(0.003)	−0.019***(0.003)	−0.019***(0.003)	−0.019***(0.003)
HHI	0.029***(0.008)	0.026***(0.007)	0.015**(0.006)	0.016*(0.008)
KKZ	−0.004**(0.002)	−0.006**(0.003)	−0.006***(0.002)	−0.004***(0.001)
CONSTANT	0.009*(0.005)	0.011*(0.006)	0.016**(0.007)	0.020**(0.008)
Time dummies	Yes	yes	yes	yes
Sargan test	18.86(0.75)	17.01(0.85)	18.75(0.78)	19.59(0.66)
AR(1)	−3.43(0.00)	−3.38(0.00)	−3.01(0.00)	−3.03(0.00)
AR(2)	−0.19(0.85)	−0.17(0.87)	−0.21(0.71)	−0.29(0.74)
Instruments	21	21	21	21
Observations	598	598	598	598
No. of banks	32	32	32	32

Note: The reported results in this table are the impact of capital and risk on bank interest margins. The dependent variable is NIM1 across all models. A higher value of NIM1 denotes a higher bank margin, and vice versa. The estimation technique is the Two-Step System GMM panel regression. 1 %, 5 % and 10 % levels significance represents by ***, ** and *, respectively. Heteroskedasticity-robust standard errors are in parentheses. Details about the variables are presented in Table 1.

However, as shown in Table 6, after replacing the alternative variables and time dummies in our estimated model, we observed no significant changes in the coefficient sign and values compared to the results reported in Table 5. This proves the main estimation based on Eqs. (2), (3), (4), (5) using the two-step GMM technique remains valid and coherent.

### 4.4 The effect of Capital and Risk on the bank interest margin: Crisis Period Analysis

Further, we assess the influence of regulatory capital and default risk on bank interest margin in the recent global financial crisis (GFC) period 2007–2009. Several past studies observed the effect of the world financial crisis on bank interest margins. Among them, Poghosyan [45] performed a separate regression regarding the effect of the world financial crisis on financial intermediation cost (interest margin) and found its positive but insignificant relationship with financial intermediation cost in low-income countries. Reasoning as a recent crisis was started in the advanced economies; thus, low-income countries' banking sector was not severely affected. Moreover, low-income countries' capital markets are underdeveloped and, in some cases, absent, which makes it a barrier to contamination from advanced economies to low-income countries. However, Islam and Nishiyama [25] tested the effect of the recent crisis on bank interest margins in South Asia and found its negative and significant impact on bank interest margins. Similarly, Amuakwa-Mensah and Marbuah [83] examine the financial crisis's impact on the Ghanaian bank interest margin and find that the average margin falls throughout the crisis period compared to the pre-crisis period. The previous studies' findings of the effect of the global financial crisis on bank interest margins in developing economies are largely inconclusive, motivating us to inspect it again to conclude.

Table 7 shows a positive and statistically significant relationship between crisis dummy and bank interest margin in the Bangladeshi banking industry. The bank interest margin was raised in Bangladesh due to the liquidity shortage during the global financial crisis period. Moreover, Bangladesh's economy is vastly dependent on garments and remittance flow from abroad. Since most of the garment-importing countries are advanced economies, their adverse shocks also affected the mode of buying payments. In that situation, banks face some difficulties in L/C collection, and most buyers become the default.

**Table 7:** The impact of regulatory capital and default risk on the bank interest margin: Crisis period analysis.

Variables	(1)	(2)	(3)	(4)
	NIM1	NIM1	NIM1	NIM1
CAR	−0.004**			
	(0.019)			
CAPD		−0.002		
		(0.434)		
CAPR			−0.001**	
			(0.035)	
ENRES				−0.008*
				(0.067)
ZSCORE	0.003***	0.004***	0.004***	0.004***
	(0.000)	(0.000)	(0.000)	(0.000)
REGDR	−0.001**	−0.002***	−0.001*	−0.002*
	(0.040)	(0.000)	(0.091)	(0.082)
Bank internal control variables	yes	yes	yes	yes
Industry-level control variable	yes	yes	yes	yes
Macroeconomic variables	yes	yes	yes	yes
Year-fixed effect dummies	yes	yes	yes	yes
CRISIS	0.0001**	0.0001*	0.0001*	0.0001*
	**(0.046)**	**(0.074)**	**(0.099)**	**(0.065)**
CONSTANT	0.061***	0.061***	0.058***	0.052***
	(0.000)	(0.000)	(0.000)	(0.000)
R-squared	0.672	0.671	0.671	0.671
F-stat (P-value)	76.31***(0.00)	75.86***(0.00)	75.98***(0.00)	76.05***(0.00)
Observations	598	598	598	598

Note: The reported results in this table are the impact of capital and risk on bank interest margins. The dependent variable is NIM1 across all models. A higher value of NIM1 denotes a higher bank margin, and vice versa. The estimation technique is the Polled panel OLS regression. 1 %, 5 % and 10 % levels significance represents by ***, ** and *, respectively. Heteroskedasticity-robust P-values are in parentheses. Details about the variables are presented in Table 1.

Similarly, in remittance, a large amount of the labor force working in advanced economies, hence the financial crisis, hamper the general flow of remittance received by banks in bad times. After all, the banks passed some hard times during the crisis, and the bank interest margin was increased. Lecturing Table 7, our variable of interest regulatory capital, default risk, and their interaction term provide a similar relationship with bank interest margin after incorporating crisis dummy in our models. That justifies our main results, which are highly suitable and eligible for policymaking and citation for future research.

### 4.5 The impact of Capital and Risk on the bank interest margin: Capital Market Crash Analysis

Two capital markets are operated in Bangladesh: the Dhaka Stock Exchange (DSE) and the Chittagong Stock Exchange (CSE). Both of the stock exchanges operated with high technology. In Bangladesh, during 2010–2011, there was a severe adverse shock in both the capital market DSE and CSE. Dramatically, the share price fall started at the end of 2010 and continued up to mid-2011. Since this dramatic shock has made an epidemic for the banking sector, we are motivated to examine the effect of the capital market crash on the banking interest margin. The results are shown in Table 8.

**Table 8:** The impact of regulatory capital and default risk on the bank interest margin: Capital market crash analysis.

Variables	(1)	(2)	(3)	(4)
	NIM1			
CAR	−0.004**			
	(0.017)			
CAPD		−0.001		
		(0.473)		
CAPR			−0.001**	
			(0.048)	
ENRES				−0.004**
				(0.046)
ZSCORE	0.003***	0.003***	0.003***	0.003***
	(0.000)	(0.000)	(0.000)	(0.000)
REGDR	−0.001***	−0.002**	−0.002***	−0.001*
	(0.000)	(0.024)	(0.000)	(0.089)
Bank internal control variables	yes	yes	yes	yes
Industry-level control variable	yes	yes	yes	yes
Macroeconomic variables	yes	yes	yes	yes
Year-fixed effect dummies	yes	yes	yes	yes
CRASH	−0.002**	−0.001*	−0.001*	−0.001**
	**(0.039)**	**(0.058)**	**(0.077)**	**(0.035)**
CONSTANT	0.058***	0.059***	0.057***	0.052***
	(0.000)	(0.000)	(0.000)	(0.000)
R-squared	0.675	0.674	0.674	0.674
F-stat (P-value)	77.40***(0.00)	77.07***(0.00)	76.96***(0.00)	77.07***(0.00)
Observations	598	598	598	598

Note: The reported results in this table are the impact of capital and risk on bank interest margins after considering the capital market crash dummy. The dependent variable is NIM1 in all models, where higher values of NIM1 denote a higher bank margin and vice versa. The estimation technique is the Polled panel OLS regression. ***, ** and* indicate significance at the 1 %, 5 % and 10 % levels, respectively. Heteroskedasticity-robust P-values are in parentheses. Details about the variables are presented in Table 1.

As shown in Table 8, the crash dummy enters a negative and significant association with the bank interest margin in the Bangladeshi banking industry. During the crash period, banks invested a lot of ideal money in the capital market rather than invest in the productive sector. Similarly, the borrower takes a lot of short-notice call money from the bank with high-interest rates to invest in the capital market rather than invest in the productive sector. As a consequence, after dropping the share price intensely during the crash period, banks, as well as borrowers, fell into greater trouble. Banks and borrowers both face a liquidity crisis. As a result, banks dishonored the depositor's check, and borrowers could not repay the bank loan; thus, the default loan rate increased severely. Finally, banking transactions became a stake, and therefore, bank margins fell. Some economists argue that one of the important reasons for the capital market crash was higher liquid investments in the bank's capital market. Because the bank is an intermediary in channeling the money from depositors to potential borrowers, and the bank may invest the long-term deposits in the productive sector to generate profit, in such a case, the bank becomes a borrower instead of an intermediary.

As shown in the reported results in Table 8, after considering the crash dummy variable in our models, the variable of interest regulatory capital, default risk, and their interaction terms provide a similar relationship with bank interest margin compared to results presented in Table 5. We have proved another way the models applied in this study fit in any situation.

Finally, Fig. 4 graphically supports a document showing that the bank interest margin decreased during the period of the capital market crash (2010–2011).

### 4.6 The impact of Capital and Risk on the bank interest margin: during and pre-Covid 19 period

The banking sector deserves special attention due to the recent pandemic and the new normal period. The present-day crisis caused by the COVID-19 pandemic has hit the world economy hard, triggering significant damage to every aspect of the global banking system, and Bangladesh is no exception [84]. During this pandemic times, emerging economies suffer a lot compared to the first economy [85].

Thus, we segregate the data into two phases: the pre-COVID period (2000–2019) and the during-COVID period (2020–2022). Since the during period of data is comparatively low and the time series panel data is quit inadequate, we thus run the Simple OLS to check the regulatory impact on the bank margin. To ensure a similar methodology, we also run the simple OLS for the pre-COVID period.

The pre-COVID (2000–2019) 20-year sample period regression under simple OLS is presented in Table 9. As can be seen, the pre-COVID period and the whole sample period show similar results. However, the intensity of impact is comparatively low in the pre-COVID period. On the flip side, the impact on the bank margin during the COVID period might be high.

**Table 9:** The impact of regulatory capital and default risk on the bank interest margin: Pre-COVID Period analysis.

Variables	(1)	(2)	(3)	(4)
	NIM1			
CAR	−0.006**			
	(0.022)			
CAPD		−0.002*		
		(0.073)		
CAPR			−0.011*	
			(0.067)	
ENRES				−0.021**
				(0.019)
ZSCORE	0.012***	0.001***	0.002***	0.002**
	(0.000)	(0.000)	(0.000)	(0.012)
REGDR	−0.024***	−0.013**	−0.003**	−0.003
	(0.000)	(0.024)	(0.029)	(0.100)
Bank internal control variables	yes	yes	yes	yes
Industry-level control variable	yes	yes	yes	yes
Macroeconomic variables	yes	yes	yes	yes
Year-fixed effect dummies	yes	yes	yes	yes
CONSTANT	0.034***	0.047***	0.021***	0.033***
	(0.000)	(0.000)	(0.000)	(0.000)
R-squared	0.591	0.624	0.589	0.547
F-stat (P-value)	68.22***(0.00)	71.11***(0.00)	69.57***(0.00)	70.54***(0.00)
Observations	502	502	502	502

Note: The reported results in this table are the impact of capital and risk on bank interest margins after considering the capital market crash dummy. The dependent variable is NIM1 in all models, where higher values of NIM1 denote a higher bank margin and vice versa. The estimation technique is the Simple OLS regression. ***, ** and* indicate significance at the 1 %, 5 % and 10 % levels, respectively. Heteroskedasticity-robust P-values are in parentheses. Details about the variables are presented in Table 1.

As shown in Table 10, during the COVID period, the impact of regulatory capital on the bank interest margin shows highly significant than in the pre-COVID period. Reasoning as, during the COVID period, the banking system stability highly suffered from a stack of money market velocity. Thus, a higher portion of earning assets become classified loans within a short period of time. During this time, regulatory capital reserves play a vital role in reducing the bank margin and the probability of default risk as well.

**Table 10:** The impact of regulatory capital and default risk on the bank interest margin: During COVID Period Analysis.

Variables	(1)	(2)	(3)
	NIM1		
CAR	−0.009***		
	(0.000)		
CAPR		−0.021***	
		(0.000)	
ENRES			−0.037**
			(0.019)
ZSCORE	0.003*	0.017***	0.007*
	(0.089)	(0.000)	(0.029)
REGDR	−0.001	−0.003**	−0.012*
	(0.240)	(0.049)	(0.096)
Bank internal control variables	yes	yes	yes
Industry-level control variable	yes	yes	yes
Macroeconomic variables	yes	yes	yes
Year-fixed effect dummies	yes	yes	yes
CONSTANT	0.032***	0.039***	0.036***
	(0.000)	(0.000)	(0.000)
R-squared	0.422	0.463	0.469
F-stat (P-value)	56.02***(0.00)	54.36***(0.00)	58.45***(0.00)
Observations	96	96	96

Note: The reported results in this table are the impact of capital and risk on bank interest margins after considering the capital market crash dummy. The dependent variable is NIM1 in all models, where higher values of NIM1 denote a higher bank margin and vice versa. The estimation technique is the Simple OLS regression. ***, ** and* indicate significance at the 1 %, 5 % and 10 % levels, respectively. Heteroskedasticity-robust P-values are in parentheses. Details about the variables are presented in Table 1.

## 5 Conclusions

This study examines the impact of regulatory capital restrictions and default risk on commercial banks' interest margins in Bangladesh. Using the two-step GMM estimation considering 32 Bangladeshi commercial banks from 2000 to 2022, we produce robust findings that regulatory capital restrictions reduce the bank interest margin. In contrast, the increasing rate of default risk induces the bank interest margin in the Bangladeshi banking sector. During the COVID pandemic period, the coefficient shows the intensity of impact was high compared to the pre-COVID period.

We use net interest margin (NIM1) as the proxy for bank interest margin and ZSCORE as the proxy for default risk. While we use four proxies of regulatory capital (CAR, CAPD, CAPR, and ENRES) to inspect the impact on bank interest margin. Moreover, we examine the second-order marginal effect of capital and risk on bank interest margin by employing the interaction of regulatory capital and default risk (REGDR). Most of the results justify our theoretical hypothesis.

Regarding the variables of interest, we find stringency in regulatory capital assists in increasing the bank interest margin, while higher default probability bound banks to charge a lower bank interest margin. Moreover, we find the interaction term of regulatory capital and default risk assist in reducing the bank interest margin.

Among the bank-specific variables, cost inefficiency and funding strength enter positive and significant with bank interest margin. Besides, management efficiency and income diversification come negative and significant with bank interest margin, reserve enters negative, but the relationship is insignificant with bank interest margin in the Bangladeshi banking sector.

Market concentration introduces positive and significant with bank interest margins, indicating that the ordinary Structure–Conduct–Performance (SCP) hypothesis quiet be present in the Bangladeshi banking sector. Among the macroeconomic variables, the KKZ index comes negative and significant with bank interest margin, indicating a vast possibility of increasing institutional quality to reduce the bank interest margin in Bangladesh. Interest rate risk shows a positive significance with bank interest margin, specifying that the volatility of money market interest rates positively influences bank interest margin. Finally, inflation enters positively and significantly with the bank interest margin, ensuring that reducing the purchasing power of money induces the bank interest margin.

Despite the several strengths of this study, we have faced some limitations regarding sample constructions. The combined sample of Islamic and commercial banks regarding bank interest margin may lose its harmony, as Islamic banks operate through 'mudharabah' instead of fixed interest rates. Moreover, the only consideration of commercial banks may not be an ideal representation of the overall Bangladeshi banking industry. The involvement of Investment, Savings, Krishi, and Bank holding companies might preview the more precise picture of relevant topics. Against this backdrop, the total number of observations decreased compared to the number of banks in Bangladesh. Regarding future research directions, we suggest incorporating some potential control variables such as information asymmetry, merger and acquisition, dividend policy, and Basel III leverage ratio. Moreover, we recommend examining the South Asian banking sector margin with our employed model.

## 6 Policy implications

Regarding the policy formulations, we suggest that the central bank, the paramount regulatory of the banking industry in Bangladesh, should ensure a full phase implementation of Basel III shortly in all types of banks in Bangladesh. Moreover, the ongoing COVID-19 pandemic creates an urgency to update Basel Accord 'Basel IV' application requirements due to the adverse shock of the world economy falling. At the bank level, we recommend that management explore diversified investment opportunities to reduce risk. Finally, in recent days, one of the crucial problems is increasing bed loan holdings by politicians. We suggest the government take careful and transparent measures to reduce the loans granted to politically connected people instead of investing in productive bucket opportunities like SME financing to women and potential micro-entrepreneurs to boost the cycle of banking channeling money.

## Availability of data and materials

Data will be made available on request.

## Funding

This research received no external funding.

## CRediT authorship contribution statement

**Munni Begum:** Writing – original draft. **Mohammed Mizanur Rahman:** Writing – original draft, Data curation, Conceptualization. **Mohammad Omar Faruq:** Writing – review & editing.

## Declaration of competing interest

The authors declare that they have no known competing financial interests or personal relationships that could have appeared to influence the work reported in this paper.

## Figures and Tables

**Table A1:** Test of Non-Stationary

Variables	χ^2^	P-Value
Bank Interest Margin (NIM1)	−17.50	0.0000
Bank Interest Margin (NIM2)	−17.55	0.0000
Regulatory Capital (CAR)	−21.99	0.0000
Regulatory Capital (CAPR)	−16.94	0.0019
Regulatory capital (ENRES)	−14.57	0.0027
Default Risk (ZSCORE)	−11.62	0.0000
Operating Cost Ratio (OPERRO)	−16.95	0.0000
Management Efficiency (MANEFF)	−12.60	0.0000
Reserves (RSVS)	−21.30	0.0000
Funding Strength (TLTD)	−12.74	0.0000
Income Diversification (INDI)	−17.85	0.0000
Market Concentration (CR3)	−14.56	0.0000
Market Concentration (HHI)	−16.18	0.0000
Governance Index (KKZ)	−10.45	0.0000
Interest Rates Risk (SDMIR)	−11.85	0.0000
Inflation (INF)	−14.94	0.0000

Note: The table represents the Augmented Dickey-Fuller (ADF) test-fisher type findings, where the alternative hypothesis of stationarity has been accepted for all the variables at 1 % level of significance. In the case of Stochastic cost inefficiency and any dummy variables, this test is not applicable.

## Footnotes

6 (1) Whether the risk-weighted capital is in the line of Basel guidelines?,(2–3) Is the minimum risk-weighted capital vibrate with each banks credit and market risk?, (4–6) whether any of the following substracted from the capital for measuring minimum capital ratio, (a) market value of loan loss not traced in financial statement, (b) unobserved losses on securities settlements, (c) Unobserved foreign exchange vibrates losses, (7) Is the revaluation gains approved as part of capital?, (8) Is the sources of capital inspect by the regulatory body?, (9) Is it allowed to increase the capital by assets other than cash or government securities?, (10) Is borrowed fund allowed as capital? The index quantifies as the value equal 1, if the questions (1–8) = yes, 0 otherwise, besides equal 1, if the questions (9–10) = no, 0 otherwise.

7 (1–8) Does the banking license given by following: (a) Formulate by law, (b) Predetermine organization chart, (c) Financial forecasts of first 3-year, (d) Information about stockholders financial condition, (e) Imminent directors background, (f) Imminent managers background, (g) New bank capitalization and their sources of funds, (h) New bank market distinction mechanism. The index formulated as the value equal 1, if the questions = yes, 0 otherwise.

8 World Development Indicators, World Bank.

## Footnotes

1 The Central Bank of Bangladesh.

2 Bank Regulation and Supervision Database.

3 https://sec.gov.bd/slaws/Risk_Based_Capital_Adequacy_Rules,_2019 (Accessed on 04/01/2022).

4 Bangladesh Bank: Annual report (July 2015–June 2016), Chapter-5 (Banking Sector Performance, Regulation and Bank Supervision).

5 48. Coleman, A.D., N. Esho, and I.G. Sharpe, *Does bank monitoring influence loan contract terms?* Journal of Financial Services Research, 2006. **30**(2): p. 177–198. explain that the banks operated with superior monitoring efforts could charge a larger cost of financial intermediation. Similarly, 49. Chen, A.H., S.C. Mazumdar, and Y. Yan, *Monitoring and bank loan pricing*. Pacific-Basin Finance Journal, 2000. **8**(1): p. 1–24. examine the U.S. branches of Japanese banks and find a positive association between monitoring activities and bank loan spreads.

9 We run the xtabond2 command developed by 71. Arellano, M. and O. Bover, *Another look at the instrumental variable estimation of error-components models*. Journal of econometrics, 1995. **68**(1): p. 29–51.

10 Price of labor = personal expenses/total assets, price of fixed assets = depreciation cost/total assets, price of fund = total interest expenses/total deposits 79. Fiordelisi, F., D. Marques-Ibanez, and P. Molyneux, *Efficiency and risk in European banking*. Journal of Banking & Finance, 2011. **35**(5): p. 1315–1326.

11 These two variables are the alternative measure of regulatory capital. Hence, two variables are not used together in one model.
